# Conventional Audiometry, Extended High-Frequency Audiometry, and DPOAE for Early Diagnosis of NIHL

**DOI:** 10.5812/ircmj.9628

**Published:** 2014-01-05

**Authors:** Amir Houshang Mehrparvar, Seyyed Jalil Mirmohammadi, Mohammad Hossein Davari, Mehrdad Mostaghaci, Abolfazl Mollasadeghi, Maryam Bahaloo, Seyyed Hesam Hashemi

**Affiliations:** 1 Department of Occupational Medicine, Shahid Sadoughi University of Medical Sciences, Yazd, IR Iran; 2 Industrial Diseases Research Center, Shahid Sadoughi University of Medical Sciences, Yazd, IR Iran

**Keywords:** Audiometry, Otoacoustic Emissions, Spontaneous, Hearing Loss, Noise

## Abstract

**Background:**

Noise most frequently affects hearing system, as it may typically cause a bilateral, progressive sensorineural hearing loss at high frequencies.

**Objectives:**

This study was designed to compare three different methods to evaluate noise-induced hearing loss (conventional audiometry, high-frequency audiometry, and distortion product otoacoustic emission).

**Material and Methods:**

This was a cross-sectional study. Data was analyzed by SPSS (ver. 19) using chi square, T test and repeated measures analysis. Study samples were workers from tile and ceramic industry.

**Results:**

We found that conventional audiometry, extended high-frequency audiometry, low-tone distortion product otoacoustic emission and high-tone distortion product otoacoustic emission had abnormal findings in 29 %, 69 %, 22 %, and 52 % of participants. Most frequently affected frequencies were 4000 and 6000Hz in conventional audiometry, and 14000 and 16000 in extended high-frequency audiometry.

**Conclusions:**

Extended high-frequency audiometry was the most sensitive test for detection of hearing loss in workers exposed to hazardous noise compared with conventional audiometry and distortion product otoacoustic.

## 1. Background

Noise is among the physical exposures originated from fluctuations in air pressure (1) and is a common exposure in many industrial settings. Noise has several effects on human health, some include: concentration disturbance, memory loss, anxiety, depressive behavior, muscular contraction, tachycardia, and hypertension (2). Noise most frequently affects hearing system, as it may typically cause a bilateral, progressive sensorineural hearing loss at high frequencies (1). Noise-induced hearing loss (NIHL) is the second most common acquired hearing loss following presbycusis, and is known as an occupational disorder long ago. It was estimated in 1981 that about 9 million workers in the US are exposed to hazardous levels of noise in the workplace (3). In spite of implementation of hearing conservation programs, NIHL is among the most important and frequent occupational disorders and the second cause of occupational injuries. NIHL is permanent and irreversible, but it can be preventable (4). Early detection of hearing loss especially before involvement of speech frequencies is of great importance.

The most common method for assessment of hearing loss is pure-tone audiometry which is performed at frequencies of 250 to 8000 Hz. This method evaluates the whole hearing pathway from external ear to the hearing cortex (5). Recently, some other methods have been proposed to detect the probability of hearing loss in an earlier time. One of the proposed methods for early diagnosis of hearing loss is extended HFA which evaluates hearing thresholds at frequencies higher than 8000 Hz (i.e. 10000, 12000, 14000, 16000, 18000, and 20000 Hz). It is believed that these frequencies are affected earlier than conventional frequencies due to exposure to noise (6-8). OAE is another method proposed by some researchers for early diagnosis of NIHL (9, 10). Several studies have been performed to assess these methods for detecting the effect of noise on hearing in an appropriate time. Most studies have found extended HFA as a sensitive test to detect the effect of noise on hearing (11-15), although some results are opposite to this hypothesis (16, 17). De Sá compared hearing thresholds in conventional and high frequencies among normal young subjects and found that the highest threshold was at 18000 Hz (18).

For the first time in 1978, Kemp recognized acoustic emissions due to the movement of outer hair cells in the cochlea (19). OAE is an objective and quick examination easily performed and does not need acoustic conditions, so it was recommended as a surrogate for audiometry (20). Porto et al. found that the frequency of hearing loss was higher at 6000 -14000Hz among noise-exposed workers (13). Somma et al. found high-frequency audiometry (10000 -16000 Hz) to change earlier than conventional frequencies (500-6000 Hz) (14). Mehrparvar et al. found that high frequencies of audiometry are more severely affected by noise than conventional frequencies (12). Some other studies also found high-tone audiometry a useful means for early detection of NIHL (11, 15). High-frequency audiometry is believed also to find hearing loss due to ototoxic drugs or substances sooner than conventional audiometry (21, 22).

Oeken et al. found a decrease in amplitude of DPOAE at 2-5 KHz frequencies after exposure to noise which was reversed after a recovery time (23). Plinkert et al. found that TEOAE is the most sensitive test for the diagnosis of TTS in comparison to DPOAE and PTA (24). Kores et al. found that DPOAE is more sensitive than PTA at low frequencies (25). Other studies have also found DPOAE as a more sensitive test than conventional audiometry for the diagnosis of NIHL (20, 26-29). There are few studies comparing OAE and HFA for early diagnosis of NIHL. Sliwinska-Kowalska assessed 17 workers exposed to noise and found 10000Hz to have the highest difference with 4 and 6 KHz, and DPOAEs were absent at 4 KHz in most individuals (30). Han et al. found DPOAE as the most sensitive test for NIHL compared to conventional and HFA (31).

## 2. Objectives

In this study we aimed to compare three different tests for early diagnosis of NIHL (conventional PTA, extended HFA and DPOAE) among noise-induced workers.

## 3. Materials and Methods

This was a prospective cross-sectional study conducted in Yazd, Iran from 15/10/2011 to 30/5/2012. Sample size was calculated based on a power of 80% and a two-sided significance level of 5%. It was calculated to be 240 subjects (120 in each group). Workers of 3 tile and ceramic factories were assessed. The subjects in the first group (exposure to noise level higher than 85 dBA) were randomly selected, and subjects in the second group (without exposure to noise) were matched to the first group regarding their age and work experience. Those older than 50 years old, with a history of acoustic trauma, history of exposure to ototoxic substances or consuming ototoxic drugs, and history of smoking were excluded from the study.

A questionnaire containing medical and occupational history was filled for each subject. Otoscopic examination was performed for all subjects, and those with abnormal tympanic membrane were excluded from the study. Conventional PTA was performed for each subject (device: audiometer AC 40, with earphone TDH 39) in an acoustic chamber meeting criteria of ANSI 2004 at the following frequencies: 500, 1000, 2000, 3000, 4000, 6000, and 8000Hz (32). Then extended HFA was performed for all subjects with the same device and earphone R80 at 10000, 12000, 14000, and 16000 Hz in the same situation by the same audiologist. OAE was elicited with the Capella (Madsen) in the fast screen mode in a quiet room by the same audiologist. DPOAE was recorded by the DPgram method. The f2/f1 ratio was kept at 1.2. The stimuli levels were kept constant at L1 = 65 dB SPL, and L2 = 55 dB SPL.We considered abnormal hearing threshold as thresholds higher than 20 dB at each frequency in conventional and HFA (33). Noise-induced hearing loss was considered as the same threshold at 3000, 4000 or 6000 Hz. Abnormal HFA was considered as more than 20 dB decrease at each frequency. Abnormal result of DPOAE was considered as lower than 10 dB response amplitude (34) which was divided into low-tone (500, 1000, and 2000Hz) and high-tone (3000, 4000 and 6000Hz) abnormal OAE. Data was analyzed by SPSS (ver. 19) using T test, chi square test and repeated measures analysis. This study was the result of a residency thesis in Shahid Sadoughi University of Medical Sciences and was approved by the ethics committee of the research vice-chancellor of this university with the number 2432 (11.8.2011). The study was performed according to Helsinki declaration, and an informed consent was obtained from each subject (in Persian).

## 4. Results

In this study, 263 subjects entered the study and 16 subjects were excluded (11 individuals had conductive hearing loss, and 5 had a history of exposure to ototoxic substances); so at last 526 ears were assessed. All subjects were males. Subjects were divided into two groups: with exposure to hazardous noise (142 subjects), and 121 subjects without any exposure. Mean exposure to noise in the first group was 91.97 ± 4.15 dBA (time weighted average-8 hours). Mean age and work experience were 35.00 ± 6.33 and 10.76 ± 5.52 years, and 34.15 ± 5.76 and 11.14 ± 6.12 years in the first and second groups, respectively. The difference between the two groups regarding age (P = 0.81) and work experience (P = 0.71) was not significant. Table 1 shows the mean hearing thresholds at different frequencies of conventional and high-frequency audiometries in the both groups. Thresholds are compared by repeated measures analysis.

**Table 1.** Mean Hearing Thresholds at Different Frequencies of Audiometry in Each Group

Frequency (Hz)	Mean (SD)		P value	
	Case (n = 142)	Control (n= 121)	Within Subjects	Between Subjects
**250**	-	-	0.032	< 0.001
Right ear	9.20 (3.01)	10.07 (1.24)	-	-
Left ear	8.99 (2.70)	10.23 (1.50)	-	-
**500**	-	-	0.61	< 0.001
Right ear	8.10 (2.50)	10.26 (1.99)	-	-
Left ear	8.02 (2.82)	10.19 (1.44)	-	-
**1000**	-	-	0.10	< 0.001
Right ear	7.73 (3.01)	10.53 (2.86)	-	-
Left ear	8.03 (3.38)	10.53 (2.58)	-	-
**2000**	-	-	0.29	< 0.001
Right ear	7.96 (3.29)	10.81 (4.14)	-	-
Left ear	7.87 (3.61)	11.11 (4.45)	-	-
**3000**	-	-	0.71	< 0.001
Right ear	8.42 (3.64)	12.96 (7.74)	-	-
Left ear	8.71 (3.92)	13.34 (7.32)	-	-
**4000**	-	-	0.14	< 0.001
Right ear	9.29 (3.19)	15.84 (10.07)	-	-
Left ear	9.51 (3.56)	17.34 (11.68)	-	-
**6000**	-	-	0.18	< 0.001
Right ear	11.05 (5.13)	19.38 (12.99)	-	-
Left ear	11.22 (5.36)	21.03 (14.13)	-	-
**8000**	-	-	0.28	< 0.001
Right ear	10.86 (5.29)	17.80 (13.49)	-	-
Left ear	10.91 (4.52)	18.15 (13.75)	-	-
**10000**	-	-	0.42	< 0.001
Right ear	5.67 (4.68)	10.00 (8.63)	-	-
Left ear	4.93 (4.25)	9.59 (7.27)	-	-
**12000**	-	-	0.93	< 0.001
Right ear	5.86 (7.51)	12.90 (9.32)	-	-
Left ear	5.65 (7.47)	12.81 (8.20)	-	-
**14000**	-	-	0.003	< 0.001
Right ear	7.35 (8.41)	19.80 (12.33)	-	-
Left ear	6.91 (8.12)	20.96 (11.68)	-	-
**16000**	-	-	0.001	< 0.001
Right ear	10.61 (11.09)	29.80 (13.69)	-	-
Left ear	9.93 (10.14)	32.71 (14.59)	-	-

**Figure 1.**
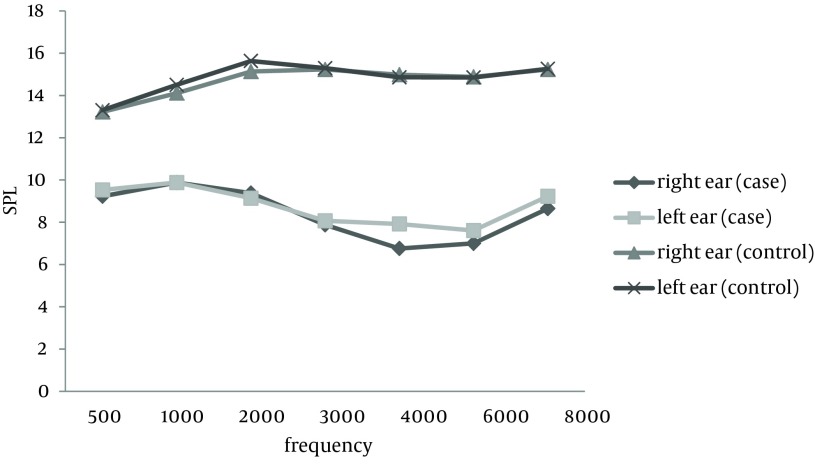
DPgram of Right and Left Ears in the Both Groups

DPOAE was significantly different in the two groups at all frequencies. Figure 1 shows the DPgram of both ears in each group.

Table 2 compares the frequency of hearing loss in conventional audiometry with abnormal high-tone audiometry and DPOAE

**Table 2.** Comparison of Frequency of Noised-induced Hearing Loss With Abnormal High-tone Audiometry and Distortion Product Otoacoustic Emissions in the Case Group

Audiometric test	Hearing Loss		
	Right Ear, No. (%)	Left Ear, No. (%)	Both Ears, No. (%)
**Conventional**	38 (29)	46 (35)	86 (33)
**High-frequency **	89 (69)	98 (76)	187 (71)
**P value (CI)**	< 0.001 (0.07-0.23)	< 0.001 (0.10-0.29)	< 0.001 (0.09-0.27)
**Conventional **	38 (29)	46 (35)	86 (33)
**DPOAE (Low frequency)**	29 (22)	36 (27)	65 (25)
**P value (CI)**	0.25 (0.82-2.51)	0.23 (0.84-2.4)	0.07 (0.99-2.77)
**Conventional **	38 (29)	46 (35)	84 (32)
**DPOAE (High frequency)**	68 (52)	67 (51)	135 (52)
**P value (CI)**	< 0.001 (0.22-0.47)	0.01 (0.31-0.84)	0.002 (0.27-0.73)
**High frequency **	89 (69)	98 (76)	187 (71)
**DPOAE (Low frequency)**	29 (22)	36 (27)	65 (25)
**P value (CI)**	< 0.001 (0.44-13.51)	< 0.001 (4.72-14.41)	< 0.001 (5.98-19.33)
**High frequency **	89 (69)	98 (76)	187 (71)
**DPOAE (High frequency)**	68 (52)	67 (51)	135 (52)
**P value (CI)**	0.008 (1.22-3.37)	< 0.001 (1.75-5.05)	< 0.001 (1.62-5.11)

## 5. Discussion

NIHL is among the most common occupational diseases. Workers exposed to noise are regularly evaluated by conventional audiometry to find cases of NIHL, an irreversible disease. Early diagnosis of NIHL or early detection of ears susceptible to the effects of noise can prevent hearing loss from extension to speech frequencies. Recently, HFA and DPOAE have been proposed as predictors of NIHL in workers exposed to noise. In this study, we compared hearing loss detected by three methods (conventional audiometry, HFA and OAE). In the current study, the frequency of hearing loss at extended HFA was significantly higher than conventional frequencies among individuals with exposure to hazardous noise, which was consistent with the results of some previous studies (11-15). Although, some studies could not show this difference (16, 17). We found 4000 and 6000 Hz, and 14000 and 16000 Hz to be the frequencies with the highest threshold after exposure to noise in conventional and extended high-frequency audiometry, respectively. The authors in another study found the same results as well (12) which was in agreement with some other studies (15, 30). Sliwinska et al. found the same frequencies in conventional audiometry, but in this study 10000 Hz showed the highest threshold in extended high-tone audiometry (30).

Turkkahraman et al. consistent with the current study found 14000 and 16000 Hz to be more sensitive to noise than other frequencies (15), but Wang et al. found hearing loss at all extended high frequencies (10000-16000 Hz) which was against the results of the current study, because in the current study 10000 Hz and 12000 Hz were frequencies with a very low frequency of abnormal hearing threshold (8). Balatsouras et al. assessed HFA in persons exposed to impulse noise, and their study did not find any statistically significant threshold difference between conventional and HFA (16). Kuronen could not find as well a significant difference between conventional and HFA among pilots, although they only assessed TTS (17). Studies on OAE are somewhat controversial. In the current study, DPOAE at high frequencies (3000, 4000, and 6000 Hz) was more sensitive to noise than conventional audiometry which was consistent with the results of some studies (20, 26-29). In comparison of conventional audiometry and low-tone DPOAE (1000 and 2000 Hz), our study failed to show a significant difference between the two methods for detection of hearing loss, but Kores et al. and Oeken et al. found low-tone OAE to be more sensitive than high-tone DPOAE and conventional audiometry for detection of hearing loss (23, 25). Plinkert found TEOAE to be more sensitive than convention audiometry for detection of TTS (24).

Now, hearing conservation programs for case finding are based on PTA. PTA is subjective, time-consuming and sensitive to surrounding noise. Therefore, there is a need for more sensitive and specific methods for early detection of NIHL. Recently, OAE has been introduced as a better predictor of occupational hearing loss, especially NIHL in workers. Attias et al. compared the results of click-evoked OAE, DPOAE and PTA for the early diagnosis of NIHL in three groups of subjects (two groups exposed to noise, and a control group without noise exposure). They showed a higher sensitivity and accuracy for OAE in the diagnosis and monitoring of cochlear status following noise exposure, which was consistent with our study (35).

To the best of our knowledge few studies have compared conventional PTA, HFA, and DPOAE. In the present study, the frequency of abnormal thresholds at high-tone frequencies of audiometry was significantly higher than DPOAE, although our cases mostly had mild hearing loss, and it is believed that the sensitivity of OAE between 10 and 50 dB SPL is low (34). Han et al. found DPOAE to be more sensitive than HFA for the early diagnosis of NIHL (31). This inconsistency may be due to the differences in the severity of NIHL; our cases mostly showed mild hearing loss and DPOAE is less sensitive in the hearing losses of less than 50dB (34). Buchler et al. found 3000, 6000, 11000 and 14000Hz as the most affected frequencies of audiometry by noise, also they found that DPOAE was most severely affected at 6000 Hz, which is in agreement with our study. They did not compare HFA and OAE (5). Sliwinska et al. assessed the three different methods (30). They found the highest hearing thresholds at 4000 and 6000 Hz in conventional audiometry consistent with our results, but the frequency which was mostly affected in HFA was 10000 Hz, which was against our results, because we found 10000 Hz to be less frequently affected by noise. These inconsistencies are probably due to technical errors, because HFA is very vulnerable to technical errors. This study had an advantage; all three methods of hearing measurement were assessed and compared simultaneously, although there were some limitations:

1. Most of the subjects had mild hearing loss in conventional audiometry in which OAE results are not reliable.
2. This study has the inherent limitation of all cross-sectional studies. To reach to this conclusion that a test would become abnormal sooner than other tests, prospective studies are required.
3. Our cases were only males, so the results cannot be extrapolated to females.
4. We could not assess 18000 and 20000 Hz frequencies because of our equipment limitations.

We concluded from the results of this study that among three different methods for assessing hearing status of noise-exposed workers, HFA is the most useful one for the early diagnosis of NIHL. To recommend performing this test for workers screening, longitudinal studies should be performed to show the decrease in hearing thresholds at high frequencies.
